# *In situ* cardiac regeneration by using neuropeptide substance P and IGF-1C peptide eluting heart patches

**DOI:** 10.1093/rb/rby021

**Published:** 2018-10-12

**Authors:** Muhammad Shafiq, Yue Zhang, Dashuai Zhu, Zongxian Zhao, Dong-Hwee Kim, Soo Hyun Kim, Deling Kong

**Affiliations:** 1 Division of Bio-Medical Science & Technology, KIST School, Korea University of Science and Technology, Seoul, Republic of Korea; 2 State Key Laboratory of Medicinal Chemical Biology, Key Laboratory of Bioactive Materials of Ministry of Education, Collaborative Innovation Center of Chemical Science and Engineering (Tianjin), College of Life Science, Nankai University, Tianjin, China; 3 Center for Biomaterials, Biomedical Research Institute, Korea Institute of Science and Technology, Cheongryang, Seoul, Republic of Korea; 4 Department of Chemistry, Center for Tissue Engineering & Regenerative Medicine, Pakistan Institute of Engineering & Applied Sciences (PIEAS), Nilore, Islamabad, Pakistan; 5 Department of Physiology & Pathophysiology, Tianjin Medical University, Tianjin, China; 6 KU-KIST Graduate School of Converging Science and Technology, Korea University, Seoul 02841, Republic of Korea

**Keywords:** cardiac patch, stem cell recruitment, myocardial infarction, electrospinning

## Abstract

Cardiovascular diseases cause huge socio-economic burden worldwide. Although a mammalian myocardium has its own limited healing capability, scaffold materials capable of releasing stem cell recruiting/engrafting factors may facilitate the regeneration of the infarcted myocardium. The aim of this research was to develop cardiac patches capable of simultaneously eluting substance P (SP) and insulin-like growth factor-1C (IGF-1C) peptide. Polycaprolactone/collagen type 1-based patches with or without SP and IGF-1C peptide were fabricated by co-electrospinning, which exhibited nanofibrous morphology. SP and IGF-1C/SP patches recruited significantly higher numbers of bone marrow-mesenchymal stem cells than that of the negative control and patch-only groups *in vitro*. The developed patches were transplanted in an infarcted myocardium for up to 14 days. Mice underwent left anterior descending artery ligation and received one of the following treatments: (i) sham, (ii) saline, (iii) patch-only, (iv) IGF-1C patch, (v) SP patch and (vi) IGF-1C/SP patch. SP and IGF-1C/SP patch-treated groups exhibited better heart function and attenuated adverse cardiac remodeling than that of the saline, patch-only and individual peptide containing cardiac patches. SP patch and IGF-1C/SP patch-treated groups also showed higher numbers of CD31-positive vessels and isolectin B4-positive capillaries than that of other groups. IGF-1C/SP-treated group also showed thicker left ventricular wall in comparison to the saline and patch-only groups. Moreover, IGF-1C/SP patches recruited significantly higher numbers of CD29-positive cells and showed less numbers of Tunel-positive cells compared with the other groups. These data suggest that SP and IGF-1C peptides may act synergistically for *in situ* tissue repair.

## Introduction

Cardiovascular diseases account for the major cause of the death worldwide, which is responsible for >17.3 million deaths annually [1]. Myocardial infarction (MI) may lead to the excessive loss of cardiomyocytes, inflammation, ventricular dilation and adverse cardiac remodeling, resulting in the development of congestive heart failure. Presently, there is no complete cure for MI and current treatment methodologies possess limited ability to regenerate the damaged cardiac muscles. Consequently, the objective of the current strategies is to find solutions for the attenuation of the progressive cardiac failure and to regenerate the infarcted myocardium, which has been accomplished by either replacing the lost cardiomyocytes with transplanted stem/progenitor cells or by harnessing the paracrine effects of stem cells [2–5]. While stem/progenitor cells have enormous potential for cardiac tissue repair and may offer distinct advantages to develop the engineered heart tissues, difficulties associated with the shortage of appropriate donors, isolation and expansion of sufficient numbers of transplantable cells, and extensive *in vitro* cell manipulations limit the therapeutic potential of the cell therapy [6, 7]. Moreover, cardiac cell therapy is hampered by the poor engraftment and significant cell death after transplantation [8]. Consequently, it is imperative to find new strategies to specifically activate the endogenous tissue repair process for MI. 

Accumulative evidences support the notion that bioactive molecules, such as stromal cell-derived factor-1 alpha (SDF-1α), vascular endothelial growth factor (VEGF), stem cell factor, fibroblasts growth factor (FGF), angiopoietin-like protein 1 (Ang-1) and granulocyte colony-stimulating factor (G-CSF) can recruit endogenous stem/progenitor cells and facilitate tissue repair *in situ* [9–13]. However, most of the above-mentioned bioactive molecules are large molecular weight proteins, which cannot be easily synthesized or incorporated into scaffold materials. Moreover, due to the absence of spatiotemporal cues as well as the short half-life of many proteins, the effectiveness of protein therapeutics may be compromised. Accordingly, scaffold materials providing spatiotemporal release of a combination of bioactive factors hold great promise for *in situ* tissue regeneration [14]. Short peptide sequences, bioactive lipids and therapeutic molecules are being investigated as a replacement or an adjuvant therapy with growth factors or stem cells, which may serve as more desirable therapeutic agents due to an economical cost, ease in processing, and better delivery.

Substance P (SP) is an undecapeptide that belongs to the tachykinin neuropeptide family and is released from the terminals of specific sensory nerves. It has been shown to recruit endogenous stem/progenitor cells toward injury site for tissue regeneration [15–20]. SP has also potentials to induce neovascularization and modulate the inflammatory response [17–20]. Moreover, in comparison to the other stem cell inducing/recruiting bioactive factors, such as SDF-1, G-CSF and VEGF, SP exhibits low molecular weight, which can be easily synthesized and incorporated into scaffold materials. Despite the ongoing research activities centered on SP, several central needs remain unmet. For example, SP can be easily degraded by the endogenous peptidases and exhibits very short half-life *in vitro* and *in vivo*, which may limit its therapeutic utilization [21, 22]. Therefore, strategies focused on enhancing the residence or presence of SP *in vivo* may be very beneficial for tissue engineering (TE) applications [23]. To cope with these limitations, SP-conjugated scaffold materials have been developed, which showed therapeutic potential in the settings of various injury microenvironments, including osteochondral defects, limb ischemia, and skin wounds rendering the use of SP of enormous potential for TE applications [16, 18, 20–22].

On the other hand, most of the transplanted cells are lost due to their poor retention and engraftment at the infarct site, which is an outcome of the hostile injury microenvironment. This may be overcome by designing cell-affinitive biomaterials or preconditioning stem/progenitor cells before transplantation. Insulin-like growth factor 1 (IGF-1) is a mitogenic and a pro-survival protein, which contains a C domain peptide (IGF-1C), (GYGSSSRRAPQT) as an active region [24]. IGF-1C peptide has been reported to promote the healing of corneal epithelial wounds [25, 26]. Previously, we developed IGF-1C peptide-conjugated chitosan hydrogels, which favored the survival and therapeutic benefits of transplanted adipose-derived stem cells [27]. Similarly, Davis *et al.* designed self-assembling peptide hydrogels containing IGF-1, which supported the growth and survival of transplanted cardiomyocytes and reduced the cell apoptosis [28].

The objective of this research was to develop cardiac patches and leverage these patches with the stem cell mobilization and recruitment potential as well as provide a supportive environment for the survival and engraftment of the recruited stem/progenitor cells. We utilized an *in situ* tissue regeneration approach in which we simultaneously mobilized endogenous stem cells to the site of the injury and provided a cell-supportive microenvironment. SP was incorporated into polycaprolactone (PCL)/collagen type 1 (Col)-based cardiac patches to promote the mobilization and recruitment of endogenous mesenchymal stem cells (MSCs) to the defective site in an acute MI model. To provide the mobilized MSCs with an environment suitable for survival and/or differentiation, we immobilized IGF-1C peptide into the developed cardiac patches. SP may recruit CD29-positive MSCs, which may either secrete paracrine factor (i.e. VEGF, FGF etc.) and participate in the cardiac tissue repair or differentiate into specialized somatic cell types, such as endothelial cells (ECs) and smooth muscle cells (SMCs). Meanwhile, IGF-1C peptide may support the recruited stem cells or cardiomyocytes and enhance their survival and retention. Our patches consist of PCL, a biodegradable polymer with wide applications in TE and collagen type 1, a component of extracellular matrix (ECM), which could also provide a conducive environment for cell survival. We used co-electrospinning from two separate spinnerets to fabricate cardiac patches. Electrospinning can afford a three-dimensional architecture, which may recapitulate the native tissue’s ECM and provide physical signals for tissue repair [29, 30]. Herein, we revealed that the use of patches containing SP and IGF-1C peptide promote heart regeneration *in vivo*.

## Experimental details 

### Materials

PCL pellets (number average molecular weight, M*n* =80 000 Da) and 1,1,1,3,3,3-hexafluoroisopropanol (HFIP) were purchased from Sigma Aldrich (St Louis, USA). Chloroform (CHCl_3_) and methanol (CH_3_OH) were purchased from Tianjin Chemical Reagent Company (Tianjin, China). Triton X-100 was bought from Alfa Aesar (London, England). SP and IGF-1C peptides (purity, ≥95%) were obtained from Peptron (Daejeon, Korea). Lyophilized collagen type 1 was a gift from the Saining Biotechnology Company (Tianjin, China).

### Fabrication of cardiac patches

The hybrid PCL/Col cardiac patches were prepared by co-electrospinning from two separate spinnerets. PCL solution (10% w/v) was prepared by using CHCl_3_ and CH_3_OH (5:1 v/v) and stirred overnight. Collagen type 1 solution (8% w/v) was prepared in HFIP at ambient temperature for 24 h. SP or IGF-1C peptides were dissolved in deionized water at ambient temperature for 24 h to obtain 2 mg/ml solution. Collagen and SP or IGF-1C peptide solutions were mixed (4:1 v/v) and stirred for another 12 h. Two 10-ml syringes were filled with PCL or collagen solution with or without peptides and connected to a 21-G blunt-ended needle. The apparatus consists of a syringe pump (Cole Parmer, Vernon Hills, IL, USA), a high-voltage generator (DWP503-1AC, Dong-Wen High Voltage power supply factory, Tianjin, China), and a rotating aluminum mandrel as a collector. The aluminum foil was wrapped around the mandrel to collect the fibers. The flow rate of PCL and collagen solution was adjusted at 2 and 0.6 ml/h, respectively. The voltage between the needle tip and the rotating mandrel was 15 and 12 kV for PCL and Col, respectively. The distance between the spinneret and the collector was set at 15 cm for PCL and collagen solutions. Membranes were vacuum-dried for 48 h before further use.

### Characterization of cardiac patches

#### Morphological analysis

Morphological analysis was carried out by using a scanning electron microscope (SEM, HITACHI, X-650, Japan, voltage 15 kV) and membranes were sputter-coated with gold and palladium.

#### In vitro transwell migration assay

The migratory response of rabbit bone marrow-MSCs (BM-MSCs) toward patches was analyzed using a Transwell migration assay following a previous method [31] (Supplementary data for the detailed method). 

#### Cell proliferation assay

Proliferation of cells on the patches was examined by using cell counting kit (CCK-8). The detailed procedure is described in the Supplementary data.

#### Cell viability assay

*In vitro* cell viability on patches was examined by performing performing [3-(4,5-dimethylthiazol-2-yl)-2,5-diphenyl tetrazolium bromide] (MTT) assay. The method is described in the Supplementary data.

##### Evaluation of the release of SP

Release studies of SP peptide from electrospun membranes were carried out by using high performance liquid chromatography (HPLC, Agilent Technologies, 1200 Series, USA) with YMC-Pack Pro C18 Column (ID, 250 × 4.6 mm, S-5 µm, 12 nm) following our previous published method [30].

### Transplantation of cardiac patches

BALB/c mice (female, 20 ± 2 g) were purchased from the Laboratory Animal Centre of the Academy of Military Medical Sciences (Beijing, China), and all animal experiments were carried out using the guidelines set by the Tianjin Committee of Use and Care of Laboratory Animals and the overall project protocols were approved by the Animal Ethics Committee of the Nankai University. During the surgery, the body temperature of the mouse was maintained at 37.5 ± 0.5°C using a heating table. The animals’ conditions such as activity, behavior, lethargy, lack of appetite, and hair texture were assessed each day. The mice were euthanized, when they exhibited significant impairments of their conditions. Mice (*n *=* *5 per group) were randomly assigned to six groups as follows: sham-operation, MI-saline, MI-patch only, MI-IGF-1C patch, MI-SP patch and MI-IGF-1C/SP patch. An acute MI model was induced by the permanent ligation of the left anterior descending (LAD) coronary artery as previously described [32]. See the Supplementary data for the detailed procedure of ligation. After ligation, either saline was injected or the patches were implanted on the epicardium by a 9-0 suture. Thereafter, the chest wall and skin were closed. Sham operations were performed on another five mice without LAD.

### Echocardiography

To evaluate left ventricular (LV) geometry and function 14 days after the surgery, echocardiography (ECG) was carried out as described in detail in the Supplementary data.

### Histological analysis

After 14 days, mice were sacrificed and hearts were removed, washed with phosphate buffered saline (PBS), and snap frozen in liquid nitrogen. Frozen sections were embedded in optimal cutting temperature medium. From each heart, the sections (thickness, 5 μm) of four levels (15 mm thick) were stained with hematoxylin and eosin (H&E) and Masson’s trichrome (MT) staining. Collagen positive areas (infarct size) and the infarct wall thickness of LV were analyzed using the computerized planimetry (Axio Vision LE Rel. 4.5 software; Zeiss, Jena, Germany).

### Immunohistochemistry

To carry out immunofluorescence staining, frozen transverse tissue sections (5 μm) of hearts (*n *=* *5 for each group) were fixed in cold acetone, dried in a fume hood and washed with 0.01 mM PBS. Sections were blocked by using 5% normal goat serum (Zhongshan Golden Bridge Biotechnology, China) for 45 min at 4°C. A 0.1% Triton-PBS was used for permeation for intracellular antigen staining, before blocking. Then the rat anti-mouse CD31 antibody (BD Pharminogen, 1:100) was added on the sections for overnight at 4°C. Afterwards, sections were washed with PBS (eight times, 5 min) and incubated with Alexa Flour 488 goat anti-rat IgG (1:200, Invitrogen) or Alexa-Fluor 488 goat anti-mouse antibodies (Invitrogen, USA) for 2 h at ambient temperature. The sections without incubation with primary antibodies were used as negative controls. The nuclei were counter-stained with 4,6-diamidino-2-phenylindole (DAPI) containing mounting solution (DAPI Fluoromount G, Southern Biotech, England).

For immunohistological detection of capillaries, sections were incubated with 0.5 mM calcium chloride (CaCl_2_) for 5 min, which was followed by the incubation with isolectin B4 antibody (1:70 dilution in 0.5 mM CaCl_2_) at 4°C overnight and RT for 1 h. Sections were washed with 0.5 mM CaCl_2_ solution (thrice, 5 min) and incubated with the Sudan black solution for 7 min at ambient temperature. After washing with PBS, the nuclei were counter-stained with DAPI. Slides were observed under a fluorescence microscope (Zeiss Axio Imager Z1, Germany). The numbers of CD31-positive blood vessels and isolectin-B4 positive capillaries were counted in 10 randomly chosen high-power fields (HPFs, 400×) of the border zone. Results were expressed as cells per HPFs. For CD29 staining, sections were incubated with integrin B1 antibody (abcam, 1:1000) overnight and stained with Alexa Flour 488 goat anti-rabbit IgG (1:200, Invitrogen) antibody for 2 h at room temperature. The nuclei were counter-stained with DAPI. In situ BrdU-Red DNA Fragmentation (TUNEL) Assay Kit (ab66110) was used to evaluate cell apoptosis following the manufacturer’s instructions. Cardiac troponin-T (cTnT; Abcam, Cambridge, Massachusetts) and DAPI were used to detect cardiac muscle and cell nuclei and slides were observed under a fluorescence microscope (Zeiss Axio Imager Z1, Germany).

### Statistical analysis

All quantitative results were obtained from at least five samples for analysis. Data were expressed as the mean ±standard error of mean. Statistical analysis was performed either by using One way ANOVA followed by Tukey’s post hoc analysis or Kruskal–Wallis test followed by Dunn’s test for multiple comparisons. A value of *P *<* *0.05 was considered to be statistically significant.

## Results

### Characterization of cardiac patches

**Figure 1:**
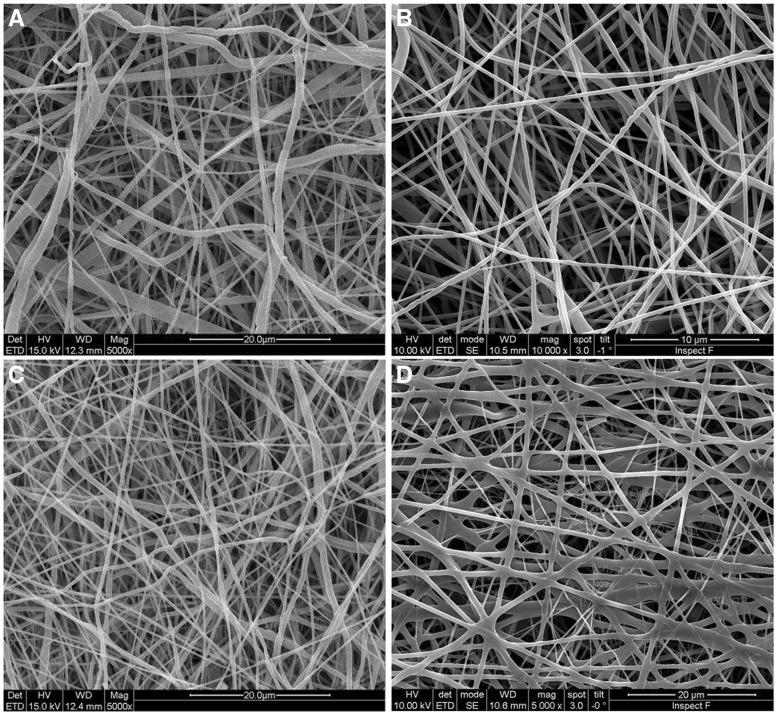
SEM micrographs of cardiac patches. (A) PCL/col, (B) PCL/col+IGF-1C peptide, (C) PCL/col+SP peptide, and (D) PCL/col+SP+IGF-1C peptide

We fabricated cardiac patches consisting of a biodegradable polymer ‘PCL’ and an ECM component ‘collagen type 1’ and incorporated SP and IGF-1C peptides to simultaneously enhance endogenous stem cell mobilization and survival, respectively. The processing parameters of PCL fibers have been optimized to fabricate electrospun membranes to facilitate cellularization and remodeling, which has been documented elsewhere [33]. The morphology of cardiac patches was assessed by SEM, which revealed the presence of uniform, continuous and smooth fibers (Fig. 1). FTIR spectra of patches revealed the presence of collagen as confirmed by its characteristics peaks (Supplementary Fig. S1). We evaluated the *in vitro* release of SP from electrospun membranes (*n *= 5 per group) by using HPLC and the cumulative released amount of SP was found to be 57.79 ± 9.96% for up to 5 days [30]. We did not observe the released amount of the peptide from electrospun membranes beyond this time point by using HPLC, which may be due to the detection limit of HPLC (<0.1 ppm) [30].

### *In vitro* transwell migration assay

**Figure 2:**
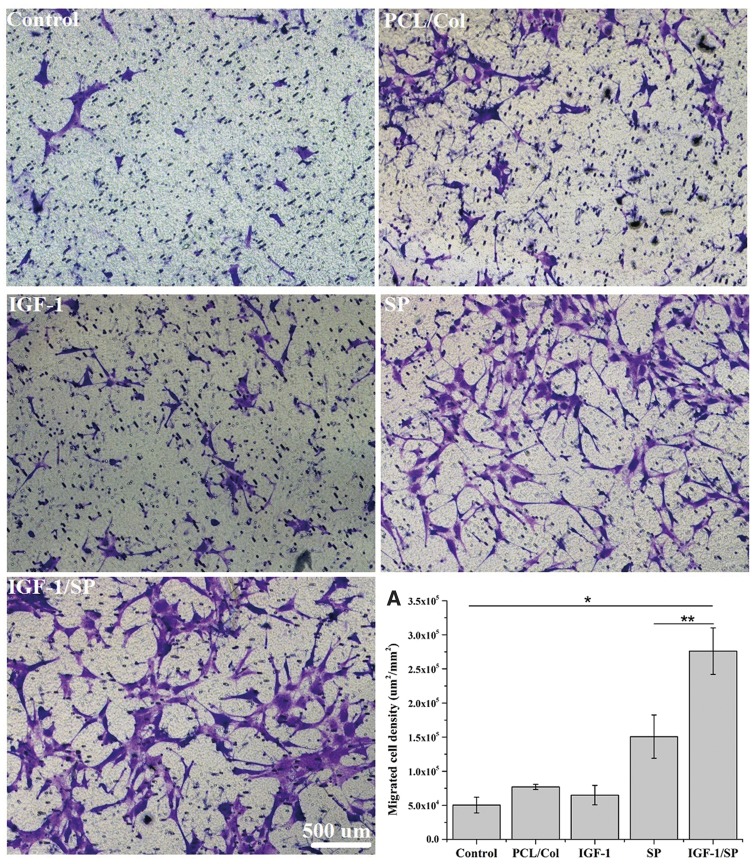
*In vitro* transwell migration assay (*n = 5* per group). Wells containing medium only served as negative controls. Negative control, patch-only, and IGF-1C patch groups recruited only few numbers of BM-MSCs. On the other hand, SP patch and IGF-1C/SP patch groups recruited significantly higher numbers of BM-MSCs than that of the other groups. The density of the recruited BM-MSCs was evaluated by using image J and found to be 50366.9 ± 11604.0, 77011.47 ± 3842.1, 64917.01 ± 14211.5, 158706 ± 31711.4 and 276112.7 ± 34117.4 µm^2^/mm^2^ in negative control, PCL/col, IGF-1, SP and IGF-1/SP groups, respectively. Results are shown as mean±SEM and evaluated by one-way ANOVA followed by Tukey’s post hoc analysis. Scale bar, 500 µm. *P < 0.05, **P < 0.01

Since SP has been shown to act as a mitogen, which enhances stem cell mobilization, an *in vitro* cell migration assay was performed to evaluate the role of cardiac patches with or without SP and IGF-1C peptides in terms of the cell migration. Wells containing medium only served as negative controls. The representative images are shown in Fig. 2. Only few numbers of BM-MSCs were migrated toward the negative control, patch-only and IGF-1C patch groups (Fig. 2). On the other hand, many cells were migrated toward SP and IGF-1C/SP patches (Fig. 2). The density of recruited BM-MSCs was evaluated by using Image J and found to be 50366.9 ± 11604.0, 77011.47 ± 3842.1, 64917.01 ± 14211.5, 158,706 ± 31711.4 and 276112.7 ± 34117.4 µm^2^/mm^2^ in negative control, patch-only, IGF-1C patch, SP patch and IGF-1C/SP patch, respectively (Fig. 2A). IGF-1C/SP patch group recruited significantly higher numbers of BM-MSCs in comparison to other groups.

### Cell proliferation assay

The *in vitro* cell growth was evaluated by using CCK-8 assay for up to 6 days. Optical density values were measured at a wavelength of 450 nm and found to be 0.3775 ± 0.0005, 0.3435 ± 0.0025, 0.3835 ± 0.0045, 0.3845 ± 0.0155 and 0.416 ± 0.004 for tissue culture plate, patch-only, IGF-1C patch, SP patch and IGF-1C/SP patch groups, respectively, at Day 3. Cell growth slightly increased at Day 6 and optical density values were found to be 0.4205 ± 0.0035, 0.365 ± 0.012, 0.4325 ± 0.0525, 0.4525 ± 0.0005 and 0.44 ± 0.0005 for tissue culture plate, patch-only, IGF-1C patch, SP patch and IGF-1C/SP patch groups, respectively. The tissue culture plate, patch-only, IGF-1C patches, SP patch and IGF-1C/SP patch did not significantly differ in terms of the cell proliferation.

### Cell viability assay

*In vitro* cell viability was examined on patches by using an MTT assay. As can be seen from the figure, patch-only group show less cell viability than that of the IGF-1C patch, SP patch and IGF-1C/SP patch for up to 72 h (Supplementary Fig. S2). Besides, IGF-1C patch, SP patch and IGF-1C/SP patch did not significantly differ in terms of the cell viability.

### Histological analysis of the cardiac patches

**Figure 3:**
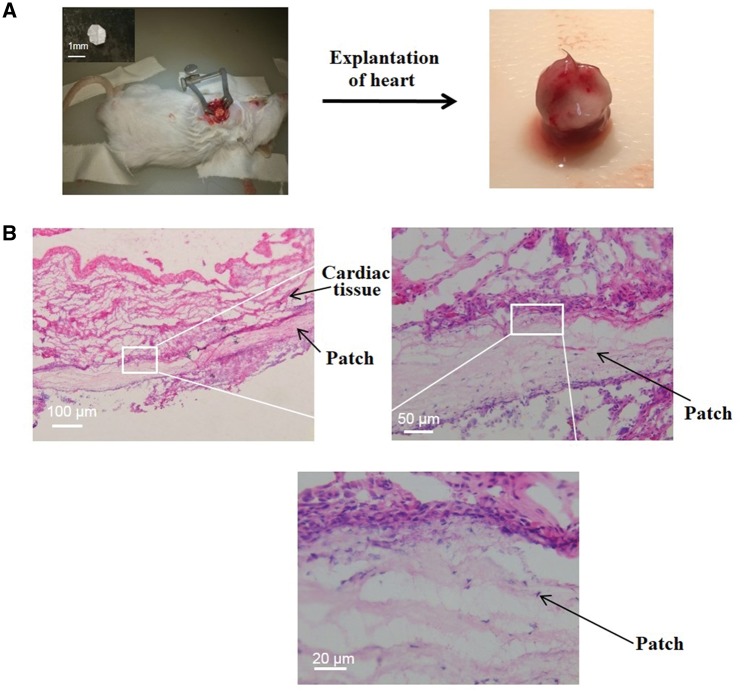
Schematic illustration of the study (A) and histological analysis of the excised hearts at Day 14 (B). (A) implantation of cardiac patches on an infarcted myocardium. Patches were sutured onto epicardium by using at least two sutures. Hearts along with the patches were harvested 14 days after transplantation for histological and immunohistochemical analysis at the tissue levels. (B) Representative H&E images showing that the implanted patches remained adhered to the myocardium and were populated by the host cells

**Figure 4:**
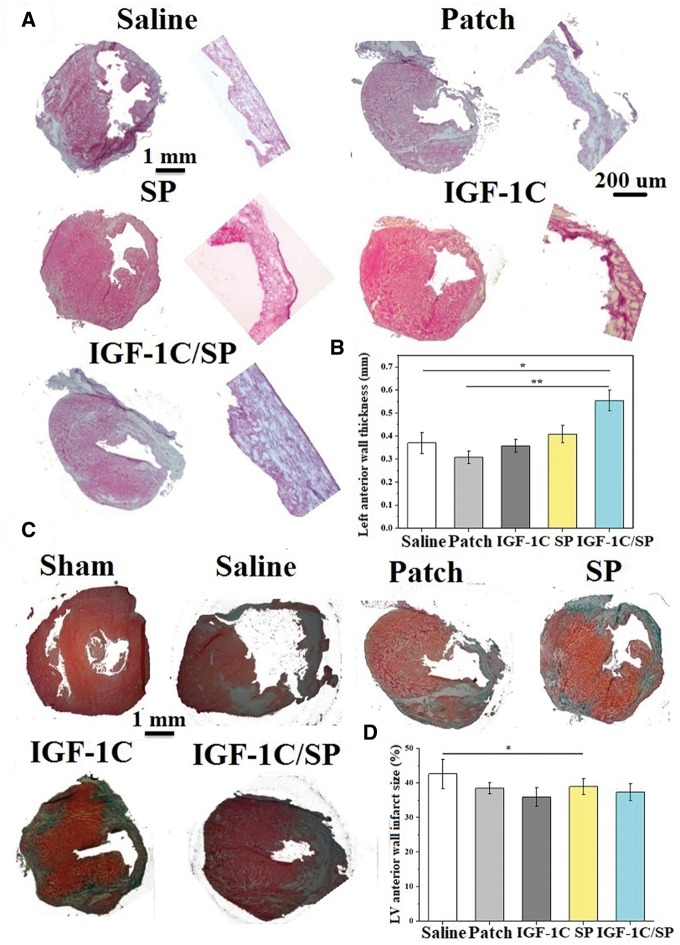
H&E and MT staining of hearts explanted 14 days after implantation. H&E staining (A), left anterior wall thickness (B), MT staining (C) and percent of anterior wall infarct size (D). Scale bars have been shown on the images. IGF-1C/SP patches exhibited significantly higher LV wall thickness in comparison to the other treatment groups. Five animals were analyzed per group. Results are shown as mean±SEM and evaluated by one-way ANOVA followed by Tukey’s post hoc analysis. (B) *P = 0.0001848 and **P = 0.00005638; (D) *P = 0.0227

The *in vivo* biocompatibility of patches was evaluated in the settings of an acute MI model in mice for up to 2 weeks. At Day 14, ECG was measured to assess the cardiac function and hearts were excised for investigations at the tissue level using histology and immunohistochemistry. The survival rate of the mice was found to be 100% in all groups following MI. Figure 3 shows the schematic diagram of the patch and its implantation in mice. H&E staining revealed that the patches remained adhered to the myocardium and were populated by the host cells (Fig. 3B). The LV wall thickness and fibrosis were evaluated using H&E staining and MT staining, respectively. The representative images of H&E and MT stained tissue sections are shown in Fig. 4. Saline, patch-only, SP patch and IGF-1C patch-treated groups did not significantly differ in terms of the LV wall thickness (saline, 0.33 ± 0.11; patch-only, 0.308 ± 0.028 mm; SP patch, 0.408 ± 0.038 mm; and IGF-1C patch, 0.358 ± 0.028 mm) (Fig. 4B). Interestingly, IGF-1C/SP patches showed significantly higher LV wall thickness than that of the saline, patch-only, IGF-1C patch and SP patch-treated groups (0.554 ± 0.0445 mm) (Fig. 4B). The infarct size was found to be 2.62 ± 4.20, 38.46 ± 1.67, 39.0 ± 2.34, 35.94 ± 2.72 and 37.34 ± 2.46% in saline, patch-only, IGF-1C patch, SP patch and IGF-1C/SP patch-treated groups, respectively (Fig. 4C and D). SP patch-treated group showed significantly less infarct size in comparison to the saline-treated group (saline vs SP patch, *P *=* *0.0227).

### Evaluation of vascularization

**Figure 5:**
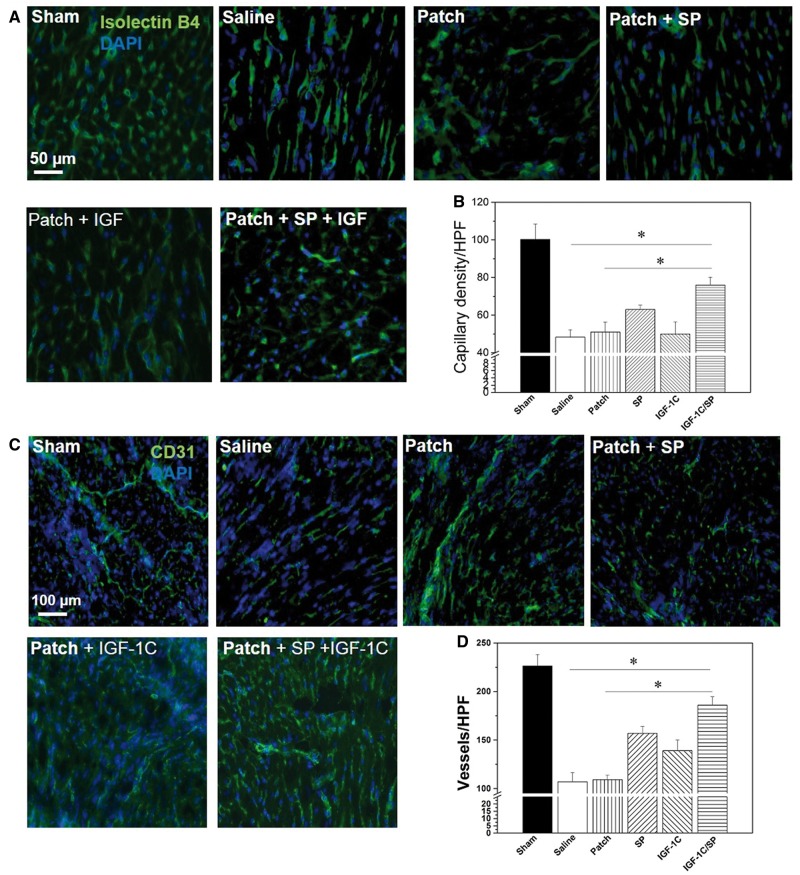
Vascularization in the explanted hearts. Excised hearts were stained with isolectin B4 (A, B) and CD31 (C, D). SP and IGF-1/SP patches showed significantly higher numbers of isolectin B4 capillaries and CD31-positive vessels. Scale bars, 50 µm (A) and 100 µm (C). SP and IGF-1C/SP patch-treated groups showed significantly higher numbers of isolectin B4-positive capillaries than that of the saline, patch-only and IGF-1C patch-treated groups. On the other hand, IGF-1C patch, SP patch and IGF-1C/SP patch-treated groups showed significantly higher numbers of CD31-positive vessels than that of the saline and patch-only groups. IGF-1C/SP-treated groups showed significantly higher numbers of capillaries and vessels in comparison to the other groups. Five animals were analyzed per group. Results are shown as mean±SEM and evaluated by one-way ANOVA followed by Tukey’s post hoc analysis. *P < 0.05

We stained sections for isolectin-B4 and CD31 to investigate vascularization (Fig. 5). After 2 weeks, SP patches showed significantly higher numbers of isolectin-B4 positive capillaries in comparison to the saline, patch-only and IGF-1C patch-treated groups (saline, 48.3 ± 4.0; patch-only, 51.0 ± 4.3, IGF-1C patch, 50.0 ± 6.5; and SP patch, 63.0 ± 2.3 capillaries per HPF) (Fig. 5B). IGF-1C/SP patch-treated group showed an increase in the isolectin-B4 positive vessels that was significantly higher than all groups (76.0 ± 4.2 capillaries per HPF). This suggests that the combination of IGF-1C peptide and SP helped improve the formation of capillaries in the infarcted myocardium.

The neo-vessels were also stained with CD31 antibody. Saline and patch-only groups did not significantly differ in terms of the numbers of CD31-positive vessels (saline, 107.95 ± 9.5 and patch-only, 109.3 ± 4.6 vessels per HPF). On the other hand, IGF-1C patch and SP patch groups showed significantly higher numbers of CD31-positive vessels in comparison to saline and patch-only groups (IGF-1C patch, 139.0 ± 11.0 and SP patch, 157.0 ± 7.0 vessels per HPF) (Fig. 5D). Interestingly, IGF-1C/SP patch groups showed many CD31-positive vessels that was significantly higher than all groups (186.0 ± 8.6 vessels per HPF) (Fig. 5D). These results reveal the formation of neo-vessels and capillaries that are most likely involved in the tissue perfusion and were plausibly resulted by SP and IGF-1C peptides.

### Evaluation of heart function

**Figure 6:**
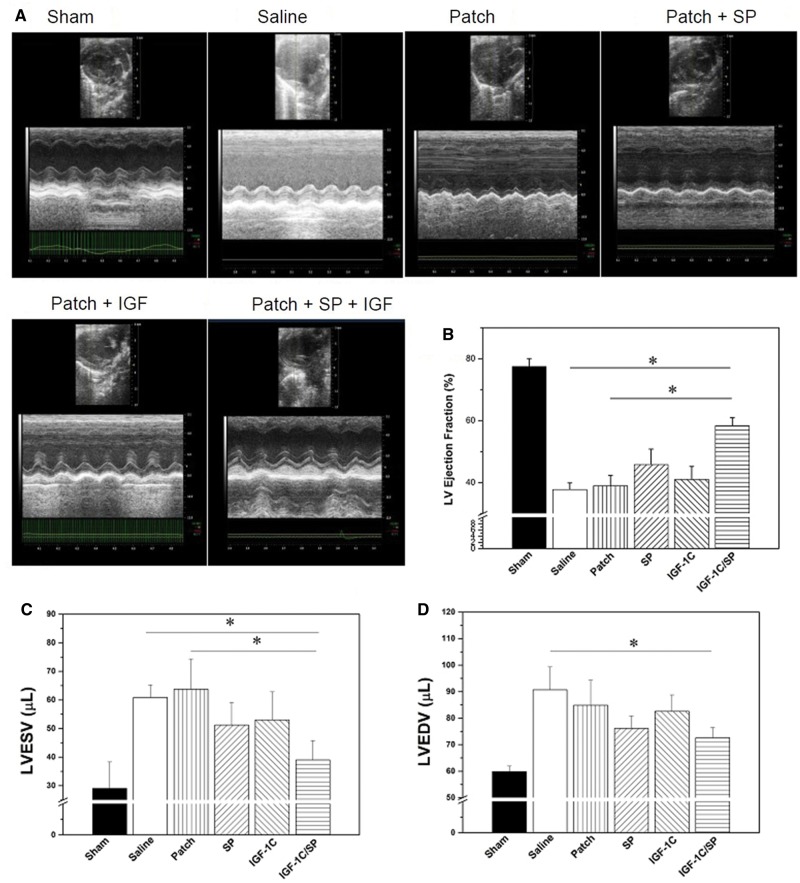
Echocardiography of mice at Day 14. (A) Representative echocardiographic 2D images of hearts of each group. Compared with the saline, patch-only and IGF-1C patch-treated groups, SP patch and IGF-1C/SP patch-treated groups showed significantly higher LVEF values (B). IGF-1C/SP patch-treated groups also displayed LVESV and LVEDV values in comparison to the other treatment groups (C, D). Results are shown as mean±SEM and evaluated by one-way ANOVA followed by Tukey’s post hoc analysis. *P < 0.05. Sham (*n* = 5), saline (*n* = 5), patch-only (*n* = 5) and peptides containing patches (*n* = 5) at 14 days

We next evaluated the *in vivo* effect of different groups comparing saline, patch-only, IGF-1C patch, SP patch and IGF-1C/SP patch. ECG was used to assess the cardiac function after patches implantation (Fig. 6). Fourteen days post-treatment, ECG analysis showed that SP and IGF-1C/SP patch-treated groups attenuated LV remodeling, which is essential to prevent heart failure following MI. No significant difference was found in the LV ejection fraction (LVEF) value among saline, patch-only and IGF-1C patch-treated groups (saline, 37.75 ± 2.11; patch-only, 38.94 ± 3.42; and IGF-1C patch, 40.99 ± 4.21%) (Fig. 6B). On the other hand, SP patch and IGF-1C/SP patch-treated groups showed significantly higher values of LVEF in comparison to the other groups (SP patch, 45.78 ± 5.01; and IGF-1C/SP patch, 58.37 ± 2.56%). Notably, IGF-1C/SP patch-treated group showed the highest improvement in the LVEF value than all groups (IGF-1C/SP patch, 58.37 ± 2.56%) (Fig. 6B).

LV end-systolic volume (ESV) and end-diastolic volume (EDV) were also measured from the ECG results. Saline, patch-only, IGF-1C patch, and SP patch-treated groups did not markedly differ in terms of the ESV values (saline, 60.86 ± 4.349; patch-only, 63.746 ± 10.603; IGF-1C patch, 53.98 ± 9.847; and SP patch, 51.22 ± 7.847µL) (Fig. 6C and D). On the other hand, IGF-1C/SP patch group showed significantly lower ESV values than that of the other groups (IGF-1C/SP patch, 39.0 ± 6.748 µL). Similarly, IGF-1C/SP patch group showed significantly less EDV values in comparison to all other groups (saline, 59.90 ± 2.055; patch-only, 84.86 ± 9.454; IGF-1C patch, 82.58 ± 6.145; SP patch, 76.147 ± 4.658; and IGF-1C/SP patch, 72.585 ± 3.875 µL) (Fig. 6C and D). The ability of IGF-1C/SP patches to improve the cardiac function after MI stresses the importance of these bioactive molecules.

### Evaluation of fibrosis

**Figure 7:**
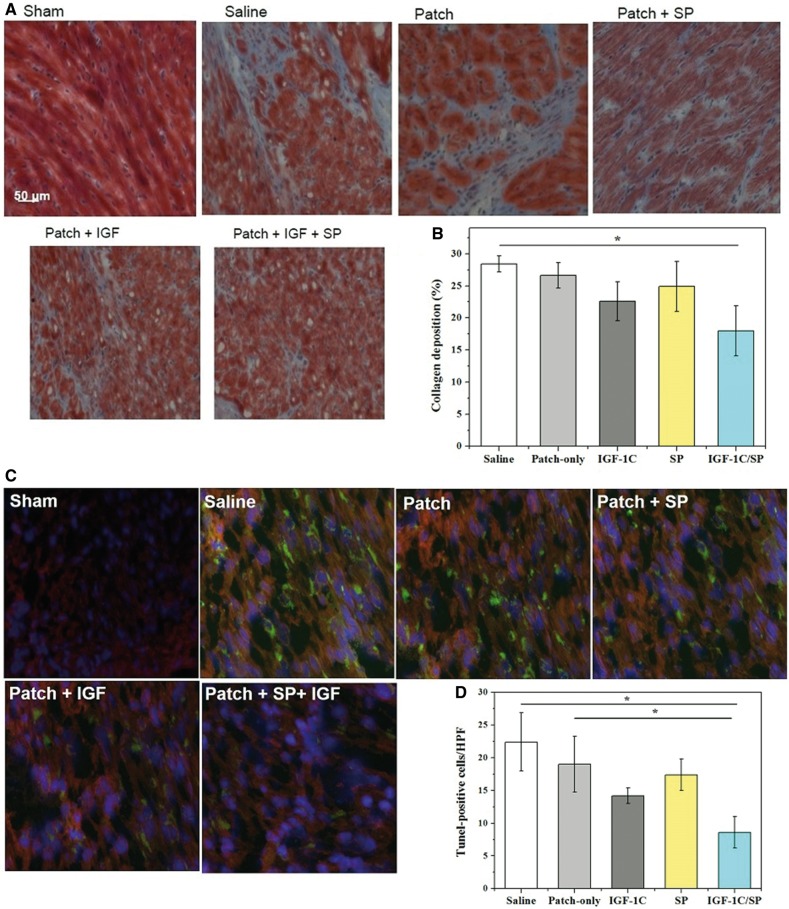
MT Staining (A, B) and Tunel assay (C, D) for evaluating fibrosis and cell apoptosis of the explanted heart tissues, respectively. IGF-1C/SP patch-treated groups showed significantly less deposition of collagen in comparison to saline-treated groups. Results are shown as mean±SEM and evaluated by one-way ANOVA followed by Tukey’s post hoc analysis. *P = 0.02351. On the other hand, saline, patch-only, SP patch and IGF-1C patch-treated groups did not markedly differ in terms of the content of tunel-positive cells, whereas IGF-1C/SP patch-treated groups showed significantly less numbers of tunel-positive cells than that of the saline and patch-only groups. Blue, DAPI; red, troponin; and green, apoptotic cells. Five animals were analyzed per group. Scale bar, 50.0 µm. Results are shown as mean±SEM and evaluated by Kruskal–Wallis test followed by Dunn’s test for multiple comparisons. *P = 0.0016

The extent of fibrosis in the different treatment groups was assessed by using MT staining. Pink muscle fibers can be distinguished from blue stained ECM. The area fraction of collagen deposition was also measured. Saline, patch-only, SP patch, and IGF-1C patch did not significantly differ in terms of the deposition of collagen (saline, 28.4 ± 7.26; patch-only, 26.64 ± 1.99; IGF-1C patch, 22.6 ± 3.07; and SP patch, 24.9 ± 3.88%). On the other hand, IGF-1C/SP patch-treated group showed significantly less deposition of collagen than that of the saline group (IGF-1C patch, 18.0 ± 3.90%) (Fig. 7A and B).

### Evaluation of cell apoptosis

Reducing cell apoptosis triggered by MI is an important goal toward recovery and repair of infarcted myocardium. Tunel assay was performed to evaluate cell apoptosis in the myocardial tissues and the representative images are shown in Fig. 7C and D. Saline, patch-only, SP patch and IGF-1C patch-treated groups did not significantly differ in terms of the numbers of the Tunel-positive cells (saline, 22.4 ± 4.45; patch-only, 19.0 ± 4.24; SP patch, 17.4 ± 2.42; and IGF-1C patch, 14.2 ± 1.17 cells per HPF) (Fig. 7D). On the other hand, IGF-1C/SP patch-treated group showed significantly less numbers of Tunel-positive cells in comparison to saline and patch-only groups (IGF-1C/SP patch, 8.6 ± 2.42 cells per HPF) (Fig. 7D). These data suggest synergistic effect of SP and IGF-1C peptide in reducing cell apoptosis after MI.

### Stem cell recruitment *in vivo*

**Figure 8:**
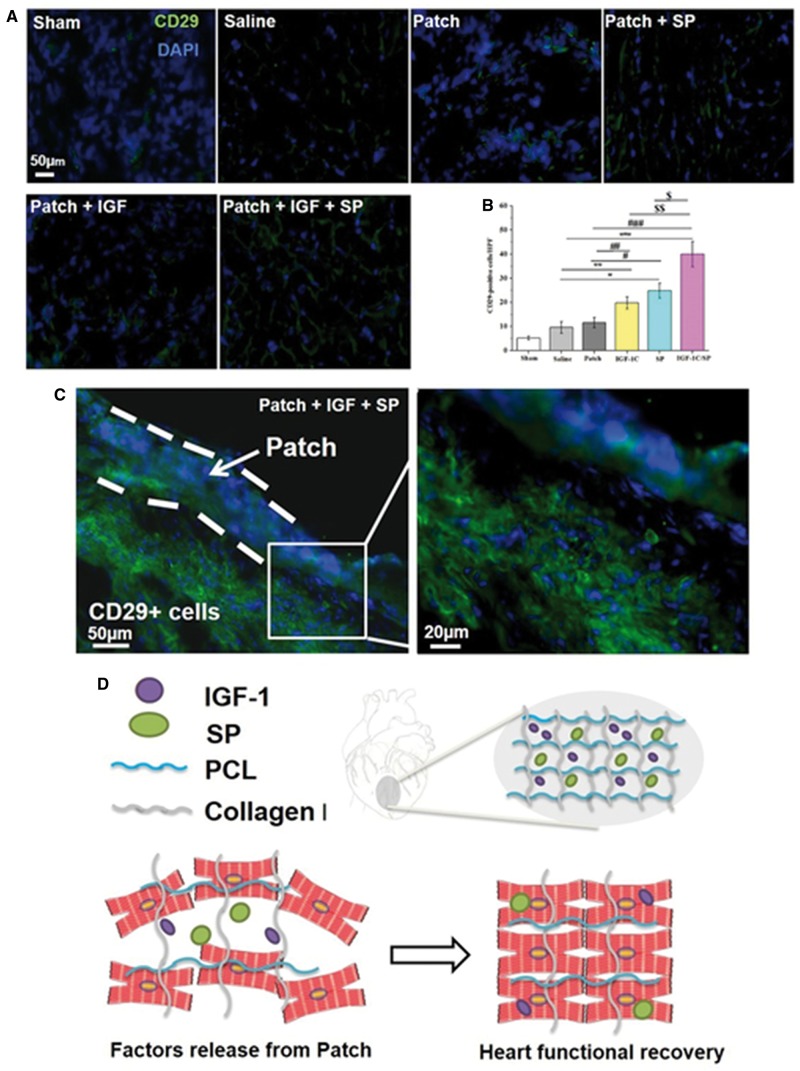
MSCs Recruitment *in vivo* and schematic illustration of the study. Excised hearts were stained with CD29 antibody (A–C). SP-eluting cardiac patches recruited significantly higher numbers of CD29-positive cells. Five animals were analyzed per group. Results are shown as mean±SEM and evaluated by one-way ANOVA followed by Tukey’s post hoc analysis. *P≤0.0001, **P = 0.0009, ***P≤0.0001, ^#^*P*≤0.0001, ^##^*P* = 0.0090, ^###^*P*≤0.0001, ^$^*P*≤0.0001 and ^$$^*P* = 0.0001. (D) Schematic diagram of the designed study. Cardiac patches containing SP alone or SP and IGF-1 peptides were fabricated by electrospinning. SP may recruit endogenous stem and progenitor cells, whereas IGF-1 peptide may enhance their retention and engraftment at the target site

SP has been documented to enhance tissue regeneration by recruiting endogenous stem and progenitor cells. Therefore, we also evaluated MSCs recruitment *in vivo* and the representative images are shown in Fig. 8. Saline and patch-only groups did not significantly differ in terms of the numbers of the CD29-positive cells (saline, 9.6 ± 2.42 and patch-only, 11.6 ± 2.15 cells per HPF) (Fig. 8). On the other hand, SP patch and IGF-1C patch-treated groups recruited significantly higher numbers of CD29-positive cells in comparison to the saline and patch-only groups (SP patch, 24.8 ± 3.12 and IGF-1C patch, 19.8 ± 2.48 cells per HPF) (Fig. 8B). Interestingly, IGF-1C/SP patch-treated group showed significantly higher numbers of CD29-positive cells in comparison to the saline, patch-only, SP patch, and IGF-1C patch-treated groups (IGF-1C/SP patch, 40.0 ± 5.25 cells per HPF) (Fig. 8B). This reflects that IGF-1C/SP may act synergistically by recruiting endogenous stem cells and supporting their survival in the infarcted area.

## Discussion

Cardiac patches manufactured by exploiting TE approaches may be able to replace the myocardial defects and may provide a solution to improve the cardiac function. Although scaffold materials can be used to provide cell-instructive cues and physical support, the modification of scaffolds is required to amplify the tissue regeneration phenomenon. Cardiac patches consisting of a variety of biodegradable components and a myriad of bioactive moieties have been pursued and shown to improve the cardiac function [34]. Moreover, different types of cells including induced pluripotent-like stem cells (iPSCs)- and embryonic stem cells (ESCs)-derived vascular cell types have been transplanted and reported to enhance the myocardial healing either by differentiation into the cardiac cell types or by the secretion of angiotrophic factors [35–37]. While appealing, exogenous stem cells need extensive *in vitro* manipulation prior to the transplantation. Additionally, transplanted stem cells hardly survive and majority of those are lost at the initial time points [35]. Besides, the usage of ESCs and iPSCs may be hampered by the ethical and teratoma formation issues.

Accordingly, recent approaches involve the activation of endogenous cells and mobilization of stem/progenitor cells from the bone marrow (BM) or the circulation. These recruited cells play a significant role in the neovascularization process. Several molecular mediators of the ischemic repair response, such as VEGF, SDF-1α, G-CSF, IGF-1, cyclin A2, ephrin B2, and Ang-1 have been shown to induce *in situ* myocardial regeneration [38, 39]. However, these are large molecular weight proteins, which cannot be easily synthesized or incorporated into scaffold materials. Alternatively, short peptide sequences, bioactive lipids and therapeutics molecules are being investigated for *in situ* tissue regeneration applications, which may be easily synthesized and incorporated into scaffold materials [15–27].

In this study, we fabricated novel cardiac patches delivering neuropeptide SP, that was shown to promote epithelialization and neovascularization in a variety of injury microenvironments by recruiting endogenous stromal like cells from the BM or circulation [15–24]. However, SP possesses short half-life and can be easily degraded by the endogenous peptidases [15, 21]. Therefore, strategies focusing at enhancing the residence or survival of SP hold great promise for SP-mediated tissue repair. Moreover, we and others have demonstrated enhanced cell engraftment and retention resulted by IGF-1 or IGF-1C peptide [9, 27, 28, 39]. Since most of the transplanted or recruited cells are lost due to a hostile microenvironment, we incorporated IGF-1C peptide along with SP to further enhance the stem cell survival and cardiac tissue repair *in vivo*.

We and others have previously reported that bioactive peptides and polymeric materials containing covalently immobilized SP can facilitate tissue repair through endogenous stem/progenitor cell recruitment [16, 18, 21, 22]. While covalent conjugation is a promising technique and may offer several advantages for tissue regeneration, the bioactivity of the peptide may be compromised because of several coupling/conjugation steps. Moreover, the lengthy and time-consuming procedure may compromise the therapeutic utilization of this approach. In this study, we fabricated cardiac patches containing either SP only or both IGF-1C peptide and SP. *In vitro* Transwell migration assay revealed that SP patches can recruit significantly higher numbers of MSCs than that of the negative and positive control groups. These results indicate that SP patches may recruit endogenous stem/progenitor cells and promote tissue repair by harnessing host’s own regenerative capabilities. The feasibility of patches was demonstrated in an acute MI model in mice for up to 14 days, in which the IGF-1/SP patch-treated group showed improved neovascularization, higher numbers of capillaries, augmented LV wall thickness, higher cardiac function, and reduced adverse cardiac remodeling than that of the saline, patch-only, or individual SP and IGF-1C peptide containing patches. Moreover, IGF-1/SP patch-treated group recruited sufficient numbers of CD29-positive MSCs and reduced cell apoptosis in comparison to the other groups. These data support the notion that endogenous stem/progenitor cells might be recruited toward bioactive patches and promote cardiac regeneration.

SP has been shown to enhance neovascularization in different types of injury models and scaffold materials, which can be due to its direct effect on ECs or its ability to recruit endothelial progenitor cells (EPCs) and stem cells [15, 17]. Amadesi *et al.* documented that BM-derived stem and progenitor cells possess neurokinin-1 receptor and are attracted by the SP gradients [17]. We and others have also previously reported that SP induces the recruitment of MSCs, EPCs, SMCs and pericytes, which then participate in blood vessel formation [15–24]. On the other hand, IGF-1C, a 12-amino acid peptide, has the similar functions of its full-length molecules [27]. IGF-1C peptide could support the cardiac cell survival and retention, attenuate fibrosis, and facilitate the recovery of heart function [27]. IGF-1 could provide an immediate strong pro-survival signal to rescue the remaining functional myocardium and reduce cell apoptosis and loss after the initial ischemic event [9, 28, 33]. Meanwhile, SP can mediate processes required for infarct repair, such as angiogenesis induction, more favorable ECM remodeling, and stem cell recruitment. Consequently, SP and IGF-1C/SP patch-treated groups led to higher capillaries regeneration in comparison to the saline, patch-only, and IGF-1C patch-treated groups. IGF-1C patch, SP patch, and IGF-1C/SP patch-treated groups also exhibited higher blood vessel regeneration compared with the saline and patch-only groups. Interestingly, IGF-1C/SP patch-treated group showed significantly higher numbers of capillaries and blood vessels than all other groups, which suggest the synergistic effect of IGF-1C peptide and SP peptide and worthy for the future investigations.

Interestingly, cardiac patch containing IGF-1C/SP significantly improved cardiac function and attenuated adverse cardiac remodeling as revealed by significantly higher LVEF values and lower ESV and EDV values in comparison to saline, patch-only, or single peptide (SP or IGF-1C) groups. Since it is well-known that neovascularization and cardiomyocytes survival plays an important role in attenuating the adverse cardiac remodeling, improvements in the cardiac function may be partly ascribed to the enhanced neovascularization as evidenced by the higher regeneration of capillaries and blood vessels in IGF-1C/SP patch-treated group in comparison to saline, patch-only or single peptide (IGF-1C and SP) containing patches [40, 41].

Besides, exogenous or endogenous stem cells have been shown to contribute to cardiac tissue regeneration by either differentiation into the vascular cell types or by paracrine mechanisms [42–45]. We also observed significant recruitment of CD29-positive MSCs in SP patch, IGF-1C patch and IGF-1C/SP patch-treated groups than that of the saline and patch-only groups. Furthermore, we observed significantly higher recruitment of CD29-positive MSCs in IGF-1C/SP patch-treated groups compared to the single peptide containing patches. Therefore, we consider that by using only one peptide may not be sufficient to produce adequate effects for regeneration in the MI model. These findings reveal that IGF-1C/SP synergism was augmented when cardiac patches incorporating the dual IGF-1C/SP therapeutic agents were transplanted into animals. These results suggest that (i) delivery of dual therapeutic factors can create benign microenvironments; (ii) recruit CD29-positive stem cells within microenvironments to form stable and vascular networks; (iii) support cardiac cell survival and reduce fibrosis.

There are also several limitations in this study. First, we implanted patches for up to 2 weeks only. Long-term evaluation of these patches will elucidate their potential for the regeneration of infarcted myocardium. Second, we did not investigate the patches at the earlier time point. Evaluation of patches at the initial time points might be helpful to clearly demonstrate the inflammatory response. Third, the exact synergistic mechanism between SP and IGF-1C leading to cardiac regeneration in MI was not elucidated in this study. Nonetheless, we elucidated that bioactive patches facilitated neovascularization, infarct stabilization, and reducing wall thinning.

## Conclusions

This study evaluated the potential of patches containing stem cell inducing/recruiting factors on *in situ* tissue repair in the settings of MI. Patches containing SP and IGF-1C peptide were successfully prepared by electrospinning. Both SP and IGF-1C/SP patches recruited significantly higher numbers of MSCs than that of the negative control, patch-only and IGF-1 groups as revealed by an *in vitro* Transwell migration assay. IGF-1C/SP patches significantly improved the heart function and attenuated adverse cardiac remodeling than that of the other groups. SP and IGF-1C/SP patches also facilitated blood vessels and capillaries formation as assessed by immunohistochemical analysis and interestingly IGF-1C/SP group showed the highest numbers of blood vessels and capillaries than that of the other groups. Moreover, IGF-1C/SP patches augmented infarct wall thickness, which was higher than that of the saline and patch-only groups. IGF-1C/SP patches also led to the less deposition of collagen compared with saline. Moreover, IGF-1C/SP patches showed the lowest numbers of Tunel-positive cells than that of saline and patch-only groups. These results suggest that these novel cardiac patches can be a good choice along with an appropriate scaffold material to enhance *in situ* blood vessel regeneration and are worthy for the future applications.

## Funding

This work was supported by the KIST Institutional Program and by the KU-KIST Graduate School of Converging Science and Technology Program. Project supported by the National Science Foundation for Young Scientists of China (Grant No. 81701839); The Youth Foundation of Tianjin Medical University (Grant No. 2015KYZQ14).

*Conflict of interest statement*. None declared.

## Supplementary Material

Supplementary Information
