# Supplementary material for: In situ cardiac regeneration by using neuropeptide substance P and IGF-1C peptide eluting heart patches
Source: Regen Biomater. 2018 Oct 12;5(5):303–16. doi: 10.1093/rb/rby021 (PMC6184517; doi:10.1093/rb/rby021)
Supplement: Supplementary Information [file rby021_supporting_information.docx]

**Supporting information**

**In Situ Cardiac Regeneration by using Neuropeptide Substance P and IGF-1C Peptide Eluting Heart Patches**

**Muhammad Shafiq, Yue Zhang, Dashuai Zhu, Zongxian Zhao, Dong-Hwee Kim, Soo Hyun Kim, Deling Kong**

***In vitro* Transwell Migration Assay**

The migratory response of rabbit bone marrow-mesenchymal stem cells (BM-MSCs) toward negative control, patch-only, IGF-1C patch, SP patch, and IGF-1/SP patch groups was analyzed using a Transwell migration assay containing a polycarbonate membrane (pore size = 8 μm; Millipore, Billerica, MA, USA) following a previous method [1]. Wells containing the medium only served as negative controls. Cells were cultured in DMEM (Life technologies, Waltham, MA, USA) supplemented with FBS (10%) and penicillin/streptomycin (1 %). Collagen type 1 (BD, Franklin Lakes, NJ, USA) (0.1 mg/ml in acetic acid) was added in the inserts and those were incubated at 37 °C with 5 % CO_2_ for 2 h. Once washed with PBS thrice, FBS-free medium (750 μL) was added in the wells of the transwell plate. Cardiac patches (diameter = 15 mm, weight = 1.5 mg) were placed in the wells. A total 300 μL of the medium was added into the inserts and BM-MSCs (3 × 10^4^ cells/well) were seeded on the top of the collagen-coated membranes. The plate was incubated at 37 °C for 48 h. Once washed with PBS, 500 μL of 4 % paraformaldehyde (PFA) was added in the inserts and wells, respectively for 20 min. PFA was removed and 500 μL of cold ethanol (100 %) was added into the inserts and wells, respectively for 20 min. Afterwards, 500 μL of crystal violet was added into the inserts and wells and removed after 15 min. The upper part of the membrane was carefully wiped with a cotton-tipped swab. The migrated cells were observed using an inverted microscope (Eclipse TE2000U; Nikon, Tokyo, Japan).

**Cell viability assay**

Cytotoxicity of Patches was evaluated by performing [3-(4,5-dimethylthiazol-2-yl)-2,5-diphenyl tetrazolium bromide] (MTT) (Sigma Aldrich, St. Louis, USA) assay. Cardiac patches were punched from electrospun membranes and sterilized using UV irradiation for 30 min. MSCs (1 × 10^4^ cells/well) were seeded into the 96-well plates. After 24h, 48h and 72h of incubation at 37°C, in a humidified 5% CO_2_ incubator, the metabolic activity of the MSCs was analyzed. Firstly, 15 μl of 5 mg/ml MTT stock solution in 1 × PBS was added into each well. After 4 hours of incubation at 37°C, in a humidified 5% CO_2_ incubator, the medium was removed and 100 µl of extraction buffer (20 µg purple crystals (Sigma Aldrich, St. Louis, USA) were dissolved in 100 μl dimethylsulfoxide (Sigma Aldrich, St. Louis, USA) was added to each well and the plates were incubated for 2 h in the dark at 37°C, in a humidified 5% CO_2_ incubator. Finally absorbance was measured at a wavelength of 550 nm and a reference wavelength of 655 nm using a microplate reader (Bio-Rad, Hercules, USA). The results were expressed as the percentage of viability relative to the control cells, which were cultured without patches treatment. Cell viability was calculated using the Equation 1.

**Equation 1:** Cell Viability (%) = (OD550-OD655) samples / (OD550-OD655) control × 100 %

**Cell proliferation assay**

Proliferation of cells on the patches was examined by using a cell counting kit (CCK-8) (Dojindo Molecular Technologies, Rockville, MD 20850, USA). Rabbit BM-MSCs were grown for up[ to 70 % confluence and harvested by using 0.25 % Trypsin-EDTA (Invitrogen). Cardiac patches (diameter, 10.0 mm) were sterilized using ethylene oxide gas at 1.0 bar and 35 °C (E.O Gas Sterilizer, PERSON-E035/50, PERSON Medical, Korea) and 40 µL of cell suspension (1.893 × 10^3^ cells) was added and the plate was incubated for up to 2.5 h. Afterwards, 300 µL of the medium was added and plates were incubated for 3 and 6 days. At specified period, 30 µL of CCK-8 was added into each well and the plate was incubated for 2.5 h. The absorbance was measured at 450 nm with UV spectrophotometer (752 Ultraviolet Grating Spectrophotometer, Shanghai).

**Ligation of left anterior descending artery**

An acute MI model was induced by the permanent ligation of the left anterior descending (LAD) coronary artery as previously described [32]. Please see the supporting information for the detailed procedure of ligation. Briefly, mice were anaesthetized through inhalation of isoflurane (1–1.5%) in O_2_, followed by intubation and mechanical ventilation using an anesthetic machine (Hallowell EMC MicroVent 1, Pittsfield, MA, USA). After the mice entered a relaxed state without breath depression and dysphoria, the LAD coronary artery was permanently ligated using a 7–0 silk suture via a left thoracotomy at the fourth intercostal space. Infarction was considered successful following the visual appearance of the pale discolouration.

**Echocardiography**

To evaluate left ventricular (LV) geometry and function 14 days after the surgery, echocardiography (ECG) was carried out on mice with a VisualSONICS echocardiographic system equipped with a 30 MHz transducer (RMV-707B; Toronto, Canada) that was equipped with Vevo MicroScan transducers (MS400). The motion of the myocardium overtime was recorded using M Mode at the level of the papillary muscle. The continuous pressure and volume signals were observed and examined using the Vevo 770 V3.0.0 Software (VisualSonics Inc., Toronto, Canada).

**Supporting Data**

**
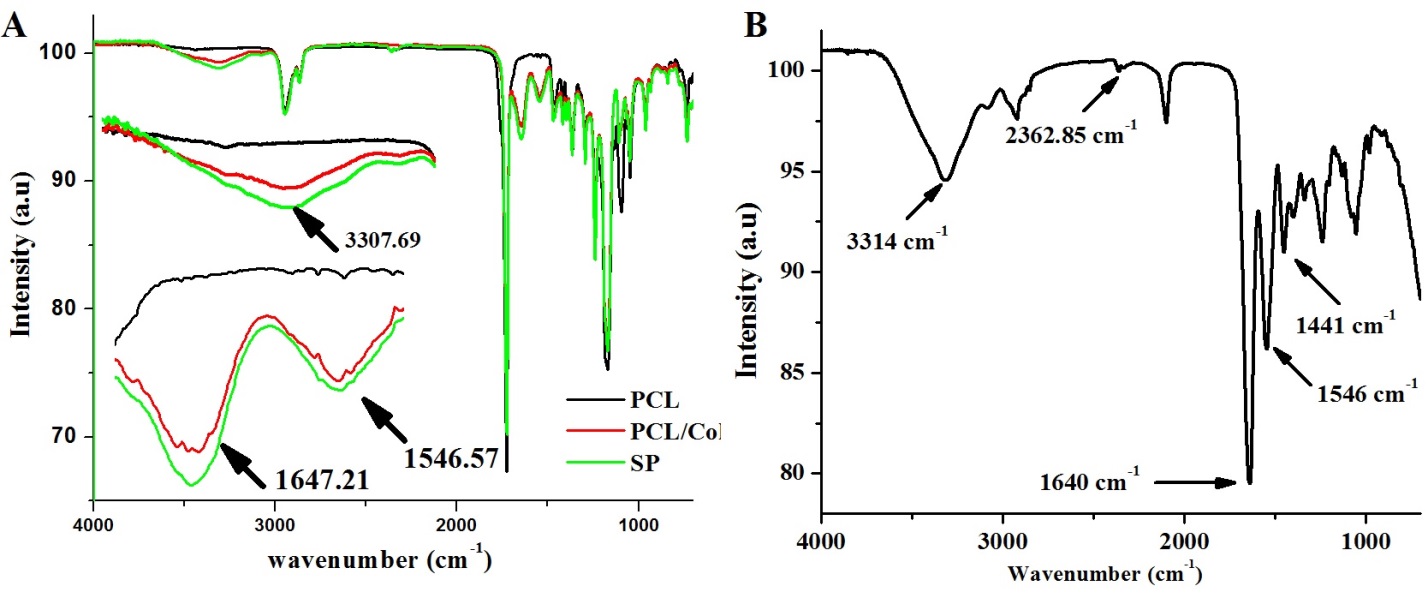
**

**Figure I.** FTIR spectra of cardiac patches. (A) PCL, PCL/Col, and PCL/Col + SP (SP group) and (B) collagen. The spectra of PCL/Col and PCL/Col + SP displayed the characteristics peaks of collagen.


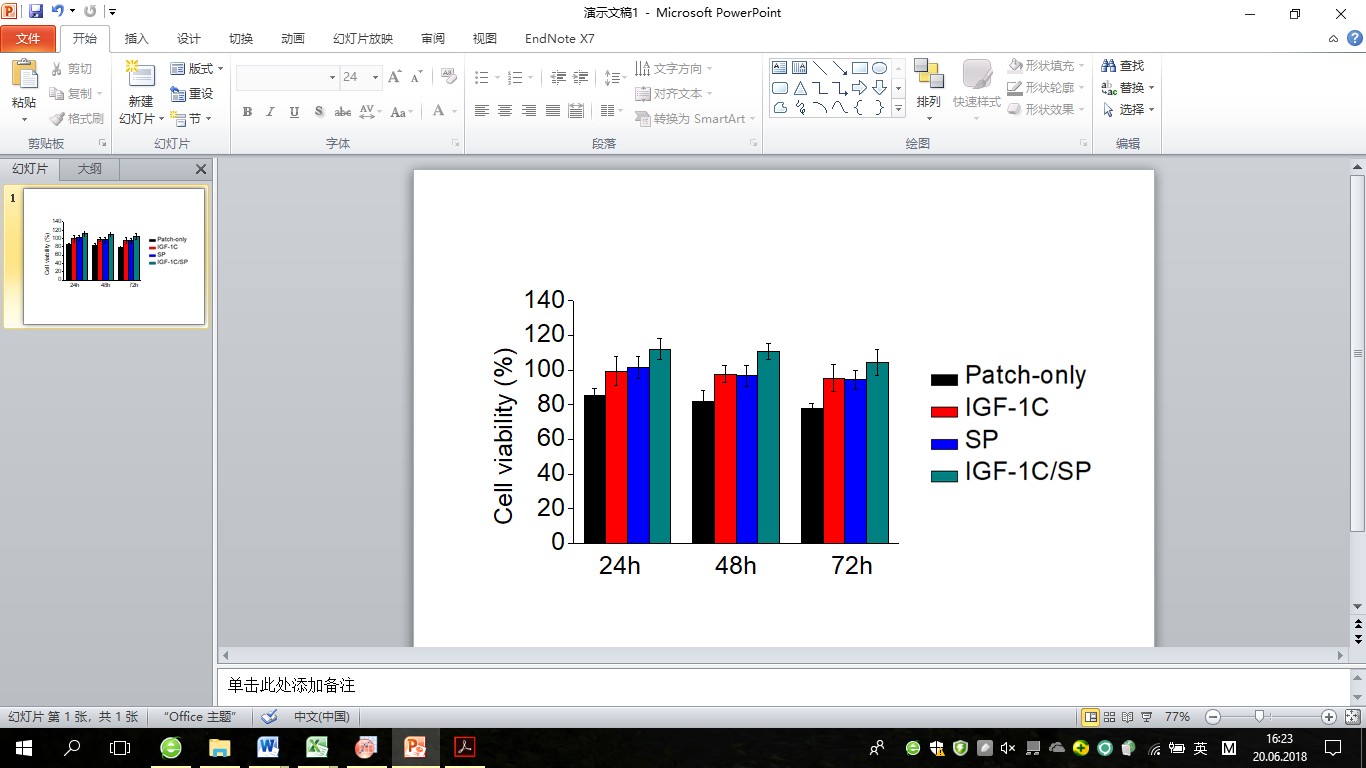


**Figure II.** Cell viability on patches for up to 72 h. IGF-1C, SP, and IGF-1C/SP patches showed higher cell viability than that of the patch-only group.

**Reference(s)**

# [1] Kim JE, Jung KM, Kim SH, *et al.* Combined treatment with systemic and local delivery of substance P coupled with self-assembled peptides for a hind limb ischemia model. *Tissue Eng Part A* 2016;22:545-55.
